# Integrated Filtration and Washing Modeling: Optimization of Impurity Rejection for Filtration and Washing of Active Pharmaceutical Ingredients

**DOI:** 10.1021/acs.oprd.3c00480

**Published:** 2024-03-12

**Authors:** Bhavik A. Mehta, Ekaterina Gramadnikova, Cameron J. Brown, Niall A. Mitchell, Sara Ottoboni

**Affiliations:** † EPSRC Future Continuous Manufacturing and Advanced Crystallisation Research Hub, c/o Strathclyde Institute of Pharmacy and Biomedical Sciences, University of Strathclyde, 99 George Street, Glasgow G1 1RD, U.K.; ‡ Siemens Industry Software Limited, 6th Floor East, 26-28 Hammersmith Grove, London W6 7HA, U.K.; § Department of Chemical and Process Engineering, University of Strathclyde, 75 Montrose Street, Glasgow G1 1XL, U.K.

**Keywords:** Filtration, washing, process modeling, qualitative optimization, impurity rejection

## Abstract

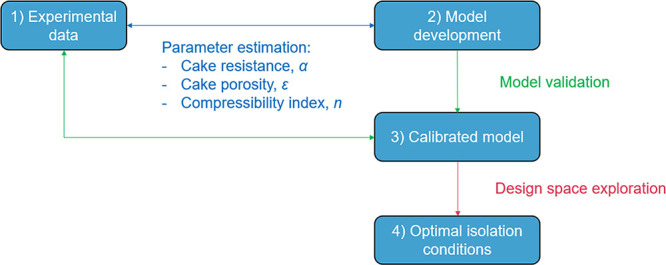

A digital design tool that can transfer material property information between unit operations to predict the product attributes in integrated purification processes has been developed to facilitate end-to-end integrated pharmaceutical manufacturing. This work aims to combine filtration and washing operations frequently using active pharmaceutical ingredient (API) isolation. This is achieved by coupling predicted and experimental data produced during the upstream crystallization process. To reduce impurities in the isolated cake, a mechanistic model-based workflow was used to optimize an integrated filtration and washing process model. The Carman–Kozeny filtration model has been combined with a custom washing model that incorporates diffusion and axial dispersion mechanisms. The developed model and approach were applied to two systems, namely, mefenamic acid and paracetamol, which are representative compounds, and various crystallization and wash solvents and related impurities were used. The custom washing model provides a detailed evolution of species concentration during washing, simulating the washing curve with the three stages of the wash curve: constant rate, intermediate stage, and diffusion stage. A model validation approach was used to estimate cake properties (e.g., specific cake resistance, cake volume, cake composition after washing, and washing curve). A global systems analysis was conducted by using the calibrated model to explore the design space and aid in the setup of the optimization decision variables. Qualitative optimization was performed in order to reduce the concentration of impurities in the final cake after washing. The findings of this work were translated into a final model to simulate the optimal isolation conditions.

## 1 Introduction

There has been a recent uptake in the use of continuous API manufacturing techniques to lower production and infrastructure costs, shorten manufacturing lead times (from a scale of months to days), and increase manufacturing flexibility and sustainability.^1,2^ Another driver is the reduction of variance in API quality.^3,4^ Particle size, habit, and purity are the typical desired product attributes to target following API purification (crystallization and isolation). Therefore, single continuous unit operations must be “smartly” integrated to enable continuous material flow from synthesis to formulation to facilitate the transition from batch to continuous manufacture.^5,6^ To do this effectively, it is essential to combine modeling, online measurement, and advanced process control approaches to predict final product quality, monitor and control processes, and reduce the risk of nonconforming products.^2,7^

Reduced material consumption during process development is another issue that the pharmaceutical industry must deal with.^8,9^ Continuous API manufacturing with a digital design provides a way to accomplish this. Digital design provides an effective way to optimize process design and cut down on time and money spent in laboratories during the development of new products. This involves the use of modeling tools to predict process performance as a function of the operating conditions for both individual unit operations as well as for integrated continuous processes. Although there are several examples in the literature of integrated continuous unit operations using flowsheet models,^10−12^ these examples primarily focus on the production of secondary drug product manufacturing processes rather than the synthesis, crystallization, and isolation of APIs.^13^

Classical isolation models view filtration and washing as two independent processes that should be modeled using two different models. The conventional cake filtration theory, which is the most frequently employed model to study dead-end filtration,^14^ describes the closing relationship, the initial and moving boundary conditions, and the relevant continuity equations.^14,15^ Further description of the existing filtration models was described in a detailed review by Wakeman et al.^16^ and Nagy et al.^17^

Rhodes^18,19^ developed a washing model that described the variables affecting the washing curve, and further studies observed different behaviors that were due to the nature of the mother liquor and wash solvent.^20^ Overall, in cases where the wettability of the solid is high with respect to the mother liquor, the wash solvent occupies the largest pores, and the mother liquor occupies the smaller ones, which can lead to two separate liquid phase networks as described by the main and side channel model.^19,21,22^ This model is used to describe the displacement-diffusion model with an axial dispersion. Another model, proposed by Svarowsky^23^ and Wakeman and Attwook,^24,25^ predicts the washing curve by considering the wash process to be driven by displacement, diffusion, and dilution washing. Finally, as reported by Tien,^21^ washing can be taken as a mass transfer process where the diffusion of the wash solvent in the mother liquor is also considered. Considering a homogeneous medium, with a uniform pore liquid flow and the diffusion dispersion effect limited in the flow direction, the concentration at the cake exit can be calculated from the following:
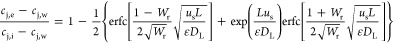
1Järveläinen and Nordén,^26^ Backhurst et al*.,*^27^ and Arora et al*.*^28^ discussed the effect of Peclet number and diffusivity coefficient on the shape of the wash curve. In modeling, the Peclet number is used as the ratio between convective and diffusion transport:
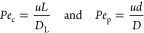
2

This work focuses on the development of a mechanistic workflow for the optimization of an integrated filtration and washing model, with the aim of minimizing impurities in the isolated cake. This work combines filtration and washing operations commonly used in API purification and isolation by combining predicted and experimental data generated during an upstream crystallization process. To validate the scenarios described using the integrated models, we selected two test compounds: mefenamic acid (MFA) and paracetamol (PCM), with a series of crystallization and wash solvents in the presence of related impurities. A combination of predicted and empirical parameters was used as the prediction input parameters. The data used for the validation stage were produced with small-scale batch pressure filter experiments. The validated model was then used to simulate an integrated filtration and washing process to maximize the purity of the isolated material through optimization. This is essential to design the isolation process capable of removing residual impurities dissolved in the mother liquor.^29^ The potential risks deriving from residual related impurities left in the isolated drug particles are well-known, like nonuniform drug content of the medicine, inconsistent drug release in the patient, or even the presence in the drug product of hazardous chemical species (e.g., carcinogens, teratogens).^30^ The isolation qualitative optimization stage is also required to minimize the residual crystallization solvent commonly responsible for particle agglomeration and lumping during the downstream drying process.^31^

The integrated modeling tool developed uses information on the product crystal suspension characteristics predicted using gPROMS FormulatedProducts to predict the filtration time, the flow rate, and the composition of the filter cake and filtrate generated during filtration. The washing of the wet filtered cake is then simulated to predict the washing efficiency and generate washing curves, cake and filtrate, and residual cake moisture content and composition of the cake.

Different washing modeling scenarios (displacement or diffusion dispersion washing) were validated to identify key process parameters (e.g., wash solvent volume and number of washes used) and their effect on filtration and washing responses. Model validation was used to identify which level of the model could describe the observed isolation data.

Overall, the objectives of the work were to

- Develop a robust model through rigorous model validation for filtration modeling, as well as both displacement and diffusion dispersion mixing during washing.
- Identify the purity of the product reached with a fixed wash ratio. The wash ratio refers to the relationship between the cake fraction and the amount of wash solvent used for each wash. Therefore, for a wash ratio of 1, the volume of solvent is equivalent to the cake void fraction.
- Conduct a design space exploration to understand the critical process parameters that affect critical quality attributes

## 2 Materials and Methods

### 2.1 MFA Case Study

The compound (MFA, 99%) and its impurities (copper(II) acetate (98%), CBA (98%), 2–3-dimethyl-*N*-phenylaniline (99%), and benzoic acid (99.5%)) were sourced from Sigma-Aldrich. The crystallization solvents used included ethyl acetate (99%, Alfa Aesar) and diglyme (99%, Alpha Aesar), whereas the wash solvents used were *n*-heptane (99%, Alfa Aesar) and cyclohexane (99%, Alpha Aesar).

The HPLC mobile phase was prepared with water (HPLC grade, VWR), ammonium phosphate (98%, Sigma-Aldrich), and ammonium hydroxide with a concentration of 3M, acetonitrile (HPLC grade, VWR), and tetrahydrofuran (99.9%, Sigma-Aldrich).

MFA, 2,3-dimethyl-*N*-phenylaniline, benzoic acid, and CBA cause serious eye damage/irritation. MFA, 2,3-dimethyl-*N*-phenylaniline, and CBA can cause skin irritation.

Diglyme, *n*-heptane, ethyl acetate, and cyclohexane are flammable solvents. Ethyl acetate causes serious eye damage/irritation. *n*-heptane and cyclohexane can cause skin irritation. Diglyme can cause damage to an unborn child and organ damage. Ethyl acetate, *n*-heptane, and cyclohexane can cause drowsiness/dizziness. Cyclohexane is toxic if swallowed. *n*-heptane and cyclohexane are very toxic to aquatic life.

### 2.2 PCM Case Study

Two grades of particle size distribution of PCM were selected to challenge different aspects of filtration, washing, and drying. The micronized material (Mallinckrodt, Inc., batch 042213E407) settles slowly and filters slowly, has a large wetted surface area to wash, and is more challenging to dry than the granular grade material.

Two structurally related compounds of PCM were used: acetanilide (A) and metacetamol (M); if present at the end of the synthesis, they could affect the crystallization process. HPLC was used to determine the purity of the isolated product. The eluents contained water (Water, ultrapure, HPLC grade, Alfa Aesar) and methanol (Methanol, ultrapure, HPLC grade, 99.8+%, Alfa Aesar), and methanol was also used as a diluent for some samples.

Three crystallization solvents were used: ethanol (purity <99.8% GC, from Sigma-Aldrich), propan-2-ol (IPA) (purity <99.5% GC, from Sigma-Aldrich), and 3-methylbutan-1-ol (known as isoamyl alcohol) (purity ≥99.5% GC, Sigma-Aldrich). As for the wash solvents, *n*-heptane (purity 99% from Alfa Aesar), isopropyl acetate (purity 99+ % from Alfa Aesar), and n-dodecane (purity 99%, from Alfa Aesar) were selected.

PCM shows oral toxicity and skin and eye irritation risks and is a skin sensitizer. Acetanilide is harmful if swallowed. Metacetamol can cause skin, eye, and respiratory irritation.

Ethanol, isopropanol, *n*-heptane, isopropyl acetate, 3-methyl-1-butanol, acetonitrile, methanol, and dimethyl sulfoxide-d are flammable solvents. Ethanol, isopropanol, isopropyl acetate, 3-methyl-1-butanol, and acetonitrile can cause serious eye damage/irritation. *n*-heptane, *n*-dodecane, 3-methyl-1-butanol, acetonitrile, and methanol can cause skin irritation. Methanol can cause organ damage (respiratory). Isopropanol, *n*-heptane, and isopropyl acetate can cause drowsiness/dizziness. Acetonitrile and methanol are toxic when swallowed. 3-methyl-1-butanol and acetonitrile can cause respiratory damage. *n*-heptane and cyclohexane are very toxic to aquatic life.

### 2.3 Methods

#### 2.3.1 MFA Case Study

From the MFA test compound, a total of 9 experiments were used for the parameter estimation (PE) and external model validation (V) of the model. The filtration and washing factors used for these experiments are listed in Table 1.

**Table 1:** Filtration and Washing Parameters Used for the MFA Experiments^a^

expt ref	PE or validation (V)	crystallization Solvent	wash solvent	isolation pressure (mbar)	wash ratio	number of washes
1	PE	ethyl acetate	cyclohexane	100	2	3
2	PE	diglyme-water	heptane	600	2	3
3	PE	ethyl acetate	heptane	600	2	2
4	PE	ethyl acetate	heptane	100	4	2
5	V	diglyme-water	cyclohexane	100	4	2
6*	PE	diglyme-water	cyclohexane	350	3	3
7*	V	diglyme-water	cyclohexane	350	3	3
8	V	diglyme-water	heptane	100	4	3
9*	V	diglyme-water	cyclohexane	350	3	3

a Experiments 6, 7, and 9 are replicas of the same filtration and washing conditions.

#### 2.3.2 PCM Case Study

A total of 9 experiments were used for the PE and validation of the model for the PCM test compound. The filtration and washing factors used for these experiments are reported in Table 2.

**Table 2:** Filtration and Washing Parameters Used for PCM^a^

expt ref	PE or validation (V)	crystallization solvent	wash solvent	solid load (%)	PCM grade	isolation pressure (mbar)	wash ratio	number of washes
1	PE	ethanol	dodecane	25	powder	800	2	2
2	PE	ethanol	dodecane	25	powder	200	2	2
3	V	isoamyl alcohol	dodecane	15	powder	200	2	2
4	PE	isoamyl alcohol	dodecane	15	micronized	800	2	2
5	PE	ethanol	isopropyl acetate	15	micronized	800	2	2
6	PE	ethanol	isopropyl acetate	15	powder	200	2	2
7*	PE	isoamyl alcohol	isopropyl acetate	20	powder	500	3	2
8*	V	isoamyl alcohol	isopropyl acetate	20	powder	500	3	2
9*	V	isoamyl alcohol	isopropyl acetate	20	powder	500	3	2

a Experiments 7–9 are replicas of the same filtration and washing conditions.

#### 2.3.3 Isolation Procedure

A modified Biotage VacMaster was used for conducting the filtration and washing of the MFA case study suspensions using manual best practice. Modifications included the accommodations for 50 mL graduated cylinders. A detailed description of the unit is reported elsewhere.^32^ The PCM case study suspensions were filtered and washed using the AWL CFD25 continuous isolation unit run in optimization mode. The CFD25 is an advanced prototype dead-end filtration unit able to filter, wash, and dry API cakes in manual, semiautomated, or even semicontinuous mode. A detailed description of the unit and the experimental procedure is reported elsewhere.^33^

##### 2.3.3.1 MFA Case Study

The MFA suspension was prepared using 2, 3-dimethelaniline, copper(II) acetate hydrate, and CBA as representative synthesis impurities. The composition of the input stream is reported in Table 3.

**Table 3:** Composition of the Input Stream for the Two Different MFA Suspensions: Ethyl Acetate and Diglyme-Water

ethyl acetate		diglyme-water	
input stream composition	mass fraction	input stream composition	mass fraction
ethyl acetate	0.876	diglyme	0.731
MFA	0.097	water	0.09
CBA	0.009	MFA	0.141
Cu (II) acetate	0.008	CBA	0.012
2,3-dimethylaniline	0.01	Cu (II) acetate	0.012
		2,3-dimethylaniline	0.015

2, 3-Dimethelaniline, copper(II) acetate hydrate, and CBA were initially dissolved in the selected crystallization solvent. The amount of MFA required to saturate the solvent solution was then added and dissolved. Finally, the amount to obtain 10% w/w solid load of MFA was added to generate the suspension. The solid phase is added to the saturated solution to mimic the slurry obtained after crystallization. In the case where the saturated solution was prepared with diglyme, a specified amount of water was added according to the synthesis liquor. For diglyme, the weight ratio between diglyme and water was 89:11.

To avoid the “antisolvent effect,” which leads to precipitation of dissolved API during the first wash step, the first stage wash was prepared using a 10:90 (V/V) mixture of pure crystallization and wash solvents, respectively. The procedure used to screen the antisolvent effect during washing is reported elsewhere.^3^ An intermediate washing stage was unnecessary since the solubility difference of MFA in 2-butanol and heptane was not critical to risk particle precipitation.

##### 2.3.3.2 PCM Case Study

The PCM suspension was prepared using 2% by mass of acetanilide and metacetamol as representative synthesis impurities. The required mass of each impurity was weighed and dissolved fully in the crystallization solvent before any PCM. The amount of PCM required to saturate the solvent solution was then added and dissolved. The last step in suspension preparation was to add the PCM required to form the cake; this PCM represents the solid load, calculated in % by mass. This two-stage addition of PCM was crucial in avoiding partial dissolution of the cake-forming particles, affecting the filter cake properties. To avoid precipitation of the dissolved active pharmaceutical ingredient during the first wash step, the first stage wash was prepared using a mixture of pure crystallization and wash solvents. The composition was selected based on the washing solvent screening methodology^33^ (Table 4). The second washing step was conducted using a pure wash solvent. In each instance, the quantity of wash solvent was based on the cake void volume and the criteria setup in the experimental design (reported in Table 2).

**Table 4:** “Antisolvent Screening” to Determine Suitable Wash Solvent Mixture for Washing 1 to Prevent Nucleation of the Particles from the Mother Liquor and Reduce the Dissolution of PCM^a^

	dodecane	isopropyl acetate
ethanol	30–70% (v/v)	30–70% (v/v)
isoamyl alcohol	20–80% (v/v)	0–100% (v/v)

a Bold format represents the percentage of pure crystallization solvent used to make the first wash solvent mixture.

#### 2.3.4 Feed Suspension Characterization

A series of raw material characterizations was conducted to investigate:

1. The particle size distribution (PSD) of the MFA material used to generate the slurry. The particle size distributions of MFA and PCM were analyzed using a wet dispersion using laser diffraction (Mastersizer 3000 laser diffraction particle size analyzer with hydrodispersion unit, Malvern Panalytical, UK). The method parameters used for the analysis of PCM and MFA were the same (Hydro MV cell, measurement duration 10 s, number of measurements 5, stabilization time 30 s, beam length 2.5 mm), but the dispersant and obscuration values were as followed:
2. PCM: particles were dispersed in isooctane, obscuration limit 5–20%.
3. MFA: particles were dispersed in heptane, with laser obscuration of approximately 15%.

Three measurements were taken for each sample. Measurements were made with and without ultrasound to detect and prevent agglomeration. Laser diffraction measurements are expressed as the volume-weighted distribution of equivalent sphere diameter. Table 5 summarizes the results from these measurements

- The solubility of MFA in the crystallization and wash solvent mixtures was predicted using COSMO*Therm* (COSMOlogic GmbH & Co. KG, Germany).^34^ The solubility of was measured with a gravimetric approach.^33^
- Calibration curves for pure MFA and CBA were gathered using a multilevel calibration method. The mobile phase for the HPLC analysis was prepared according to European pharmacopeia.^35^ An Agilent 1260 Infinity II system with a diode array and RI detector was used. The column was an Agilent Eclipse Plus C18, 4.6 × 250 mm, 5 μm, P/N 959990-902 operated at 25 °C, with a flow rate of 1 mL/min. The injection volume was 10 μL, wavelength: 254 nm, the mobile phase was 23:20:7 of acetonitrile:buffer solution:THF. Calibration curves for 2,3-dimethylaniline and cooper(II) acetate were not determined as the two compounds appeared to be insoluble in the mobile phase.
- Impurity content in the filter cake and filtrate was measured using the HPLC analytical technique. Calibration curves for pure PCM, acetanilide, metacetamol, and orthocetamol (an impurity present in the raw PCM) were gathered using a multilevel calibration method. An Agilent 1260 Infinity II system with diode array and RI detector was used. The column was an Agilent Poroshell 120 EC-C18 4.6 × 100 mm 4 μm operated at 40 °C, with a flow rate of 1 mL/min. The injection volume was 5 μL, wavelength: 243 and 230.5 nm, the mobile phase was 80% water and 20% methanol.

**Table 5:** Distribution of the Particle Size and Sphericity of the Raw PCM and MFA Compounds

PCM micronized grade		PCM powder grade		MFA	
*x*_10_ (μm)	11.1	*x*_10_ (μm)	16.6	*x*_10_ (μm)	39.03
*x*_50_ (μm)	31.7	*x*_50_ (μm)	69.8	*x*_50_ (μm)	86.95
*x*_90_ (μm)	195	*x*_90_ (μm)	198	*x*_90_ (μm)	176.48
*D*[4,3] (μm)	34.48	*D*[4,3] (μm)	77.36	*D*[4,3] (μm)	94
sphericity s_50_	0.629	sphericity s_50_	0.4127	sphericity s_50_	0.4680

#### 2.3.5 Characterization of Isolated Material

Offline sample characterization followed a precise sequence to prevent destruction of material required for further characterization.

- Cake resistance and media resistance and filtration flow rate. Data were collected manually for the MFA case study, measuring the time required to collect a series of filtrate volumes removed during filtration. The cake and filtrate masses were weighed at the end of each batch experiment. For the PCM case study, the resistance of the cake and medium and filtration flow rate were measured using the AWL CFD25 vision system.^33^
- The impurity content in the filtrates and cake was determined using the HPLC quantitative method.

### 2.4 Model Development

The integrated filtration and washing models were generated using gPROMS FormulatedProducts v2.3.1.

The integrated filtration and washing model were developed in 3 stages:

1. Filtration is modeled as a batch process, using a pressure filter model described elsewhere.^36^ Filtration stops at dryland, leaving the cake pore saturated with mother liquor. Dryland refers to a filtration stopping point where there is theoretically no mother liquor left on the surface of the cake (i.e., the free liquid height is zero)
2. Filtration and washing are modeled using a pressure filter model, where washing stages are done after filtration to dryland. The wash aliquots were introduced, respectively, as a one-off liquid charge at a specified time, as observed experimentally. One of the assumptions used in stage 2 is that the process that governs washing is displacement of mother liquor. Another assumption considered for the washing model is that no changes in the solid phase are considered (no particle dissolution or growth).
3. Washing is simulated with a mixed-suspension, mixed-product removal (MSMPR) crystallizer model under well-mixed liquid phase conditions to mimic diffusion dispersion, operating in semibatch mode. The assumption considered for the washing model is that no changes in the solid phase are considered (no particle dissolution or growth).

The equation used for the filtration and customized wash model is described below.

#### 2.4.1 Filtration Model

Dead-end filtration used in this work has been described elsewhere.^36^ The filtering process was simulated using the gPROMS FormulatedProducts v2.3.1 pressure filtration model, where the Carman–Kozeny theory was used to evaluate the resistance of the cake. The filtration process was modeled considering the initial conditions as reported in Table 6.

**Table 6:** Initial Filter Conditions Selected for the MFA Case Study Performed with gPROMS

initial conditions	unit measure	MFA	PCM
equipment and operation^a^			
media resistance	1/m	1.05 × 10^7^	1.00 × 10^8^
filter diameter	mm	27	24
driving force	mbar	450	800
equipment volume	mL	50	100
sphericity^b^	–	0.4680	0.6361
cake porosity^c^	–	0.44	0.44
initial cake mass	g	0	0
settling index			
initial conditions			
mass solid phase	g	4.34	16.25
mass liquid phase	g	43.4	48.75
crystallization solvent liquid phase mass fraction	–	0.93	0.86
solute liquid phase mass fraction	–	0.07	0.13
wash solvent liquid phase mass fraction	–	0	0
filtration temperature	°C	25	25
particle mean size	μm	94	61
particle size distribution standard deviation	μm	174	77

a Equipment geometry is equivalent to the Biotage unit system.^32^ The driving force applied is a set value in the range of driving force applied during filtration and washing processes done with the Biotage unit.

b Empirically estimated from particle size analysis.

c Empirically estimated.

Filtration was modeled as a batch process, assuming an initially uniform suspension of slurry, with cake formation occurring dynamically as a result of filtration and particle settling. The filtering process was simulated to end at dryland.

#### 2.4.2 Washing Model

The equations used for the displacement and for the diffusion-dispersion washing models were described in Section 3.2.3 by Ottoboni et al.^36^

#### 2.4.3 Model Validation, Qualitative Optimization, and Design Space Exploration

Two sets of validations were performed for the filtration model to estimate:

- The media resistance and the Carman–Kozeny cake resistance parameters, and the porosity based on initial guesses are calculated from experimental data.
- The compressibility index, where data are available.

The estimation of these parameters is essential for the comparison of simulated and experimental filtration performances and therefore to determine the goodness of the model to fit the experimental data. The estimated parameters will then be used to validate model approaches 1, 2, and 3. Furthermore, the diffusion dispersion model developed was used to explore the design space of the isolation process and identify the critical process parameters to obtain high purity levels in the final cake.

Two different design space exploration approaches were executed using the Global Systems Analysis entity in gPROMS FormulatedProducts v2.3.1. In the first approach, the first wash is set as the most significant washing process, and the aim of the design space exploration is to model the volume/time required to deliver a final solution with low levels of impurity. This approach allows for the identification of the ideal amount of wash solvent to be used during the first wash to maximize purity. The second approach puts more emphasis on the second/third wash cycles and their effect on impurity removal. This was important to understand the effect and difference between using one large wash and using multiple smaller washes with respect to the cake purity.

## 3 Results and Discussion

### 3.1 Experimental Results

#### 3.1.1 MFA Case Study

During Biotage filtration and washing experiments done with MFA test compounds, cake resistance, medium resistance, and filtrate flow rate were measured. The results of cake and medium resistance are reported in Table 7.

**Table 7:** Experimental Results of MFA Filtration and Washing Results (Experiments Settings Are Given in Table 1)^a^

experiment number	crystallization solvent	wash solvent	driving force (mbar)	cake resistance (m/kg)	medium resistance (1/m)
1	ethyl acetate	cyclohexane	100	1.23 × 10^8^	3.48 × 10^9^
2	diglyme-water	heptane	600	4.73 × 10^8^	7.39 × 10^9^
3	ethyl acetate	heptane	600	1.84 × 10^9^	1.35 × 10^10^
4	ethyl acetate	heptane	100	9.84 × 10^7^	2.98 × 10^9^
5	diglyme-water	cyclohexane	100	9.24 × 10^8^	1.21 × 10^9^
6*	diglyme-water	cyclohexane	350	1.01 × 10^8^	3.96 × 10^9^
7*	diglyme-water	cyclohexane	350	6.54 × 10^8^	3.19 × 10^9^
8	diglyme-water	heptane	100	6.69 × 10^8^	1.85 × 10^9^
9*	diglyme-water	cyclohexane	350	1.46 × 10^8^	4 × 10^9^

a *A replicate experiment to estimate variance.

Comparable values of cake and media resistance were measured for the different samples. Slightly higher cake and medium resistances were observed for experiments 2 and 3, where the highest driving force was used (600 mbar). As reported by Darcy,^37^ cake resistance and medium resistance are correlated with the driving force used.

#### 3.1.2 PCM Case Study

During the AWL CFD25 filtration and washing experiments carried out with PCM test compounds, cake resistance, medium resistance, and filtrate flow rate were measured. One of the requirements to process material with the height of the AWL CFD25 is that the cake should be at least a 30 mm height. Since the selected dose could not generate a tall enough cake, two equal doses of slurry were used. The approach the unit uses to filter two doses of slurry is the following:

- Fill the first dose of slurry in the filtration chamber.
- Filter the first aliquot of slurry.
- Feed a second aliquot of slurry on top of the first filtered cake.
- Filter the second aliquot of slurry on top of the first cake.

The results of cake and medium resistance are reported in Table 8, where measured during the first filtration.

**Table 8:** Results of the Experimental Filtration and Washing of PCM (Experiment Settings Are Given in Table 2)

experiment number	crystallization solvent	wash solvent	suspension solid load (wt %)	solid phase grade	cake resistance (m/kg)	medium resistance (1/m)
1	ethanol	dodecane	25	powder	8.98 × 10^8^	–1.24 × 10^9^
2	ethanol	dodecane	25	powder	1.45 × 10^8^	2.80 × 10^9^
3	isoamyl alcohol	dodecane	15	powder	5.63 × 10^8^	–3.4 × 10^8^
4	isoamyl alcohol	dodecane	15	micronized	4.79 × 10^9^	–4.93 × 10^9^
5	ethanol	isopropyl acetate	15	micronized	3.50 × 10^8^	–6.64 × 10^9^
6	ethanol	isopropyl acetate	15	powder	1.75 × 10^9^	–3.24 × 10^9^
7	isoamyl alcohol	isopropyl acetate	20	powder	1.14 × 10^9^	–4.2 × 10^9^
8	isoamyl alcohol	isopropyl acetate	20	powder	1.69 × 10^9^	3.38 × 10^9^
9	isoamyl alcohol	isopropyl acetate	20	powder	9.78 × 10^8^	4.92 × 10^9^

The highest cake resistance was measured during experiment 4, while the lowest cake resistance was measured in experiment 2.

This trend is explained by the nature of the suspension that was used. As reported by Ottoboni et al*.*,^32^ cake resistance values are dependent on the suspension solid load, solid phase particle size distribution, and nature of the crystallization solvent. The dependence of the solid loading is an indirect effect, as this impacts the thickness of the cake. With a thicker cake, particles at the bottom pack tighter together, leading to a variation in the filtrate flow velocity. Experiment 1 indeed uses the highest solid load; however, the use of powder PCM (larger particles and the reduced span) and the use of ethanol as a crystallization solvent cause the formation of a cake with relatively low cake resistance, compared to the others. On the other hand, experiment 2 used a suspension generated with micronized PCM (large span and smaller particles) with a weight reduction of 5% less as a solid load when compared to experiment 1, and isoamyl alcohol as a crystallization solvent. Therefore, it is observable how the nature of the solvent and solid phase particle size distribution are the main factors affecting cake resistance and filtrate flow. The negative resistance values calculated during these experiments can be inferred from the initial hold time set before the beginning of filtration. During these experiments, suspensions were left settling for 20 s to allow the vision system to track the liquid and solid to reach the phase and stop filtration to dryland. During this settling time, part of the cake was deposited on the filter media; when filtration started, the measured media resistance values were altered by the extra resistance of the presettled portion of the cake.^32^

### 3.2 Parameter Estimation

In the first instance, a parameter estimation was done to identify the particle (sphericity), cake (porosity and compressibility index), and filtration characteristics (medium resistance) to use to fit the experimental filtration performance. These estimated parameters were then used to simulate filtration and washing using the two modeling approaches—with different mixing mechanisms, which were displacement or diffusion dispersion—and to compare which model approach gave the most accurate cake composition after filtration and washing compared to the experimental data.

As reported in Methods section, two isolation model approaches were used to study the effect of different washing mechanisms: pure displacement and diffusion dispersion mechanisms. The continuous pressure filter model was used to simulate filtration stopped at dry land, followed by a pure displacement washing mechanism. Instead, the MSMPR washing model was used to simulate a diffusion dispersion washing mechanism, where washing feed information (cake composition) is provided by a decoupled Carman–Kozeny filtration model, where filtration is stopped at dryland.

#### 3.2.1 MFA Case Study

Table 9 displays the results from the parameter estimation for the MFA case studies performed by using the batch pressure filter model with filtration stopped at dryland. Four different cases were studied using the same crystallization and wash solvent combinations and the same filter characteristics. These investigations allowed us to estimate the cake and filtration properties to use for model validation and optimization. One of the estimated parameters, cake compressibility, is defined as the ability of the cake to be squeezed by the driving force applied during the filtration step. The equation used to calculate the compressibility index is reported elsewhere.^38,39^ In general, cake compressibility is calculated as the slope of the linear fitting natural logarithm of different cake resistance values, with respect to the natural logarithm of the driving forces used to determine those cake resistances. The literature reports three different levels of cake compressibility that are defined based on the value of n^40^: low and moderately compressible, *n* < 1, high compressible, *n* > 1, and extremely compressible, *n* ≫ 1. The border between high and extreme compressibility is not well-defined, but *n* values for highly compressible solids are typically reported in the interval of 1–244. Pharmaceutical cakes are generally low to moderately compressible, making them fit within the Darcy law validity range for the compressibility index. Therefore, the models were also used to determine whether the estimated values fitted the Darcy law compressibility index range.

**Table 9:** Estimated Cake and Filtration Parameters for the Different MFA Case Systems (Experiment Settings Are Given in Table 1)

crystallization solvent	wash solvent	expt ref	Carman–Kozeny sphericity	cake porosity	medium resistance (1/m)	compressibility index	objective function
diglyme-water	heptane	2	0.526	0.694	1.31 × 10^8^	0.833	112.00
diglyme-water	cyclohexane	6	0.4964	0.5258	1.31 × 10^7^	0	–93.98
ethyl acetate	heptane	3,4	0.4134	0.4804	1.6 × 10^9^	1.312	20.31
ethyl acetate	cyclohexane	1	0.399	0.476	1.46 × 10^9^	0	–42.50

In general, the estimated cake and filtration parameters using the cake and filtration parameters that match the experimental cases reported in Table 9 show good fit with the experimental data. The objective function indicates how good the fit is with experimental data: the lower the objective function, the better the model predictions. Overall, the objective functions observed here prove that the simulated compressibility value estimated for the systems with cyclohexane as the wash solvent was zero. This may be due to the cake being incompressible or because the data were not sufficient to estimate the compressibility of the cake. The other two systems estimated compressibility indices from the simulation within the Darcy law range.

#### 3.2.2 PCM Case Study

Table 10 displays the results from the parameter estimation for the PCM case studies performed using the batch pressure filter model with filtration stopped at dryland. High objective function was obtained for these simulations. The reason for these values is related to the experimental approach used to create the cake experimentally. To get the right cake to operate the AWL unit (minimum 12 mL) the machine dispensed a first dose of 60 mL of suspension, run the filtration, applied it on top of the first cake with a second dose of suspension, and then filtered the total material in the filtration chamber. Cake porosity is a fixed value derived from previous measurements.^32^ The value of the medium resistance was set as an arbitrary value, estimated from previous experimental activity.^32^ In general, the same outcomes observed for MFA were also observed for PCM. The estimated cake and filtration parameters using cake and filtration parameters show a good fit with the experimental data. Overall, the parameter estimation showed a high objective function. The simulated compressibility values estimated for the systems with isopropyl acetate as the wash solvent were zero. Like the MFA case reported above, this may be due to the cake being incompressible or the fact that the data were not sufficient to estimate the compressibility of the cake. The other two systems estimated compressibility indices from the simulation within the Darcy law range.

**Table 10:** Estimated Cake and Filtration Parameters Estimated for the Different PCM Case Systems (Experiment Settings Are Given in Table 2)

crystallization solvent	wash solvent	expt ref	Carman–Kozeny sphericity	cake porosity	medium resistance (1/m)	compressibility index	objective function
ethanol	dodecane	1,2	0.676	0.44	1.00 × 10^8^	0.321	1142
isoamyl alcohol	dodecane	4	0.691	0.44	1.00 × 10^8^	0.61	10300
ethanol	isopropyl acetate	5,6	0.636	0.44	1.00 × 10^8^	0	5422
isoamyl alcohol	isopropyl acetate	7	0.328	0.44	1.00 × 10^8^	0	7042

### 3.3 Model Validation

#### 3.3.1 MFA Case Study

The continuous pressure filter and MSMPR washing models were validated using the 9 experiments reported in Table 1 for the MFA test compound. The filtration and washing data used for the model comparison with the experiments are the filtration Darcy plot (volume of filtrate removed vs time), the solvent mass removed during filtration, and the concentration of MFA and 2,3-chlorobenzoic acid removed during washing, dissolved in the removed filtrate.

**Figure 1:**
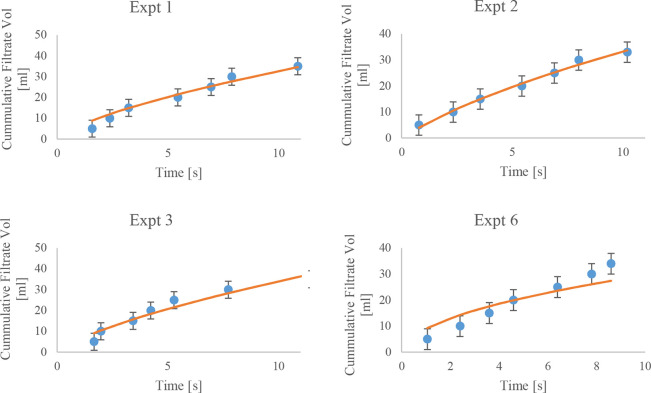
Cumulative experimental volume (blue circle) and simulated (orange line) of liquid phase removed during filtration for experiments 1, 2, 3, and 6. Filtrate volume variance corresponds to ±0.5 mL.

In general, the simulated Darcy plots reported in Figure 1 reproduce with good accuracy when compared to the experimental results, especially for Experiments 1, 2, and 3. Less precision is observed for experiment 6, which can be attributed to errors in manually collecting the experimental data. All other simulations showed reasonable fits with respect to the experiments. For more information, please see the Supporting Information.

Table 11 summarizes the experimental versus predicted results from the models. The mass of filtrate removed during Experiments 1, 2, and 6 is slightly higher compared to the predicted value. This discrepancy can be correlated to human error in accurately detecting dryland and therefore stopping the experiment. As reported by Ottoboni et al.,^32^ to stop filtration at dryland during a manual experiment done with the Biotage unit, the operator needs to manually close the valve that blocks the flow of the filtrate, precisely stopping the experiment when the liquid level reaches the top layer of the sedimented cake. There is a good probability that for these two experiments, the operator stopped the filtration experiment when the liquid level slightly surpassed the cake level (cases 1 and 4) or a layer of liquid was left on top of the cake (case 2). However, the displacement model provides an accurate filtration end point: filtration ends when the free liquid height is equal to zero, corresponding exactly to the cake height. The impurity concentration for DMA is not considered as this was too low to be measured experimentally with HPLC analysis; however, it is included in the Global Systems Analysis below to understand the trend of both impurities under different conditions. The simulated results are available in the Supporting Information.

**Table 11:** Comparison between the Experimental Data for MFA and Simulated Data Obtained with the Pressure Filter Model and with the Crystallizer Model^a^

experiment number	solvent mass left filtration (g)	solvent mass left wash 1 (g)	MFA concentration wash 1 (g/g)	CBA concentration wash 1 (g/g)
experimental data				
1	4.66	1.36	1.42 × 10^–3^	1.79 × 10^–3^
2	4.01	1.19	2.12 × 10^–2^	2.33 × 10^–3^
3	3.73	0.96	1.21 × 10^–3^	2.48 × 10^–3^
6	5.55	1.36	2.06 × 10^–2^	4.74 × 10^–3^
displacement model				
1	1.93	1.73	1.10 × 10^–4^	9.92 × 10^–5^
2	7.18	1.22	1.54 × 10^–3^	1.39 × 10^–3^
3	2.06	1.63	1.47 × 10^–4^	1.33 × 10^–4^
6	3.55	0.01	2.28 × 10^–4^	3.47 × 10^–5^
diffusion dispersion model				
1		2.74	8.30 × 10^–4^	7.47 × 10^–4^
2		7.82	2.46 × 10^–3^	2.22 × 10^–3^
3		0.47	1.11 × 10^–3^	1.00 × 10^–3^
6		4.53	7.47 × 10^–3^	1.14 × 10^–3^

a The values correspond to the crystallization solvent mass removed during filtration and the concentration of MFA and CBA removed during the first wash stage. Experiment settings are given in Table 1.

The same approach to determine the amount of filtrate removed during washing is used for the experimental data and simulated values. Instead, to experimentally determine the concentration of the dissolved species in the removed filtrate, a quantitative HPLC analysis of the filtrate was conducted. The experimental solute concentrations were compared with the simulated mass fraction of solute species removed by using pure displacement or diffusion dispersion washing mechanisms. Overall, the displacement model is not able to predict the composition well enough due to the washing mechanism approach used and its assumptions. In general, the amount of filtrate predicted with the displacement model is comparable to that of all of the experiments for filtration and washing. However, for the displacement model simulation, a consistent discrepancy is observed between the experimental and the simulated concentrations of the dissolved species removed during the first washing stage. The displacement washing mechanism assumes mechanical displacement of the mother liquor of the cake. Pure displacement is rarely achieved in a physical washing process; therefore, the residual mother liquor is always left in the small pores of the cake.^23^ To obtain a better simulated washing efficiency, in terms of mother liquor and impurity removal, it is important to simulate washing as the combination of displacement, diffusion, and dispersion mechanisms.^28^ Indeed, the diffusion dispersion model shows better accuracy in simulating the concentration of solute species (MFA and CBA) removed during washing. On the other hand, the diffusion dispersion model is unable to predict liquid mass. This is due to the semibatch operation and hold-up specifications used, leading to no outflow of filtrate or accumulation of solids in the vessel, leading to a higher predicted volume of filtrate.

#### 3.3.2 PCM Case Study

The continuous pressure filter and MSMPR washing models were validated using the 4 experiments reported in Table 1 for the PCM test compound. The filtration and washing data used for the model comparison with the experiments are the filtration Darcy plot (volume of filtrate removed vs time), the solvent mass removed during filtration, and the concentration of the PCM and acetanilide removed during washing, dissolved in the removed filtrate.

**Figure 2:**
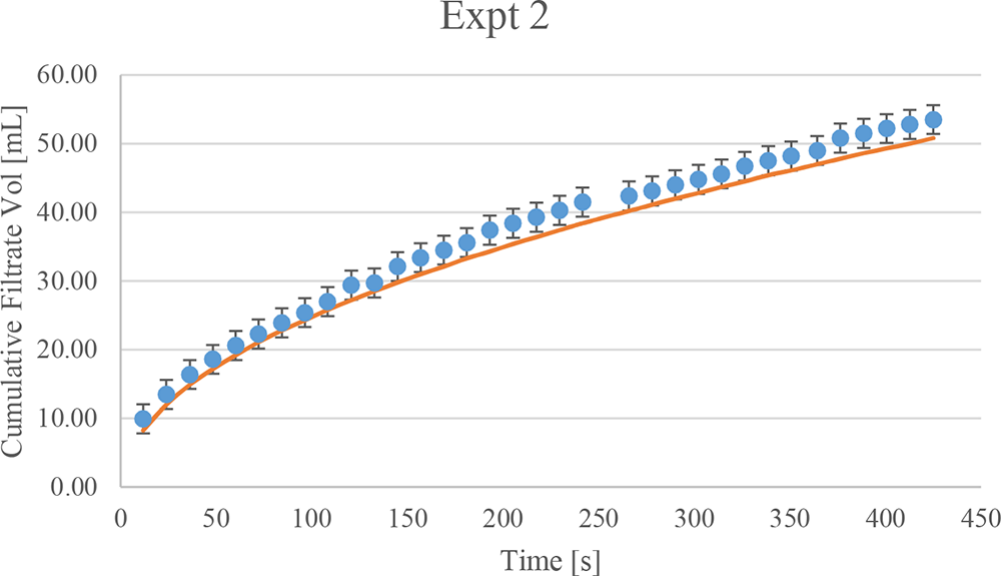
Cumulative experimental (blue circle) and simulated (orange line) volumes of liquid phase removed during filtration for experiment 2. Variance filtrate volume corresponds to ±0.5 mL.

In general, the simulated Darcy plots reported in Figure 2 reproduce with good accuracy the filtration flow rate. All other simulations showed reasonable fits with respect to the experiments. For more information, please see the Supporting Information.

Table 12 summarizes the experimental vs predicted results from the models. Four results are shown here, one for each crystallization-wash solvent combination. The impurity concentration for metacetamol is not considered, as this was not measured experimentally with HPLC analysis. Global Systems Analysis below does include metacetamol trends to show the capabilities of the model to predict impurity concentrations based on available data. The AWL CFD25 unit allows one to filter and wash aliquots of suspension and to collect the filtrate removed after each stage to characterize the composition of the filtrates and the final composition of the cake after washing with HPLC. The mass of the solvent left after filtration can be determined from the mass balance calculation (i.e., the difference between the mass of suspension filtered per each aliquot and the mass of filtrate removed during filtration). However, with this equipment, it is not possible to measure the solvent mass left after the first wash because this is an intermediate stage of the isolation process and the cake cannot be sampled before the second wash. The mass of the filtrate removed during the filtration experiment is comparable to the predicted values obtained with the displacement washing model (pressure filter). Therefore, as discussed also in the MFA case study, the displacement model provides an accurate end point. No comparison is possible for the residual filtrate left in the cake after the first wash since no experimental data were collected.

**Table 12:** Comparison between the Experimental Data and Simulated Data Obtained with the Pressure Filter Model and with the Crystallizer Model^a^

experiment number	solvent mass left filtration (g)	solvent mass left wash 1 (g)	PCM concentration wash 1 (g/g)	concentration wash 1 (g/g)
experimental data				
1	49.2		5.24 × 10^–4^	2.62 × 10^–4^
4	47.1		2.71 × 10^–2^	1.97 × 10^–3^
6	56.8			
7	41.9		2.49 × 10^–2^	1.25 × 10^–3^
displacement model				
1	44.0	8.63	4.00 × 10^–2^	2.70 × 10^–3^
4	44.8	8.82	2.21 × 10^–2^	1.17 × 10^–3^
6	54.5	9.20	4.80 × 10^–2^	1.80 × 10^–3^
7	43.5	9.77	9.05 × 10^–2^	8.42 × 10^–4^
diffusion dispersion model				
1		21.2	4.72 × 10^–2^	2.36 × 10^–3^
4		22.5	2.68 × 10^–2^	1.41 × 10^–3^
6		19.9	5.31 × 10^–2^	2.04 × 10^–3^
7		38.5	1.36 × 10^–2^	1.27 × 10^–3^

a The values correspond to the crystallization solvent mass removed during filtration and the concentrations of PCM and acetanilide (A) removed during the first wash stage. Experiment settings are given in Table 2.

Instead, to experimentally determine the concentration of dissolved species in the removed filtrate, quantitative HPLC analysis of the filtrate was conducted during wash 1. Except for experiment 1, as reported for the MFA case study, the diffusion dispersion washing mechanism is capable of predicting the composition of the filtrate removed during wash 1 with a higher accuracy when compared to the pure displacement washing mechanism. Indeed, the diffusion-dispersion model shows better accuracy in simulating the concentration of solute species (PCM and A) removed during washing.

### 3.4 Design Space Exploration

Design space exploration was done to determine which parameters affect the impurity removal during washing. Ottoboni et al*.*^32^ reported that the volume and nature of the wash solvent used, and the number of washes performed greatly affect the final purity of the cake.

#### 3.4.1 MFA Case Study

**Figure 3:**
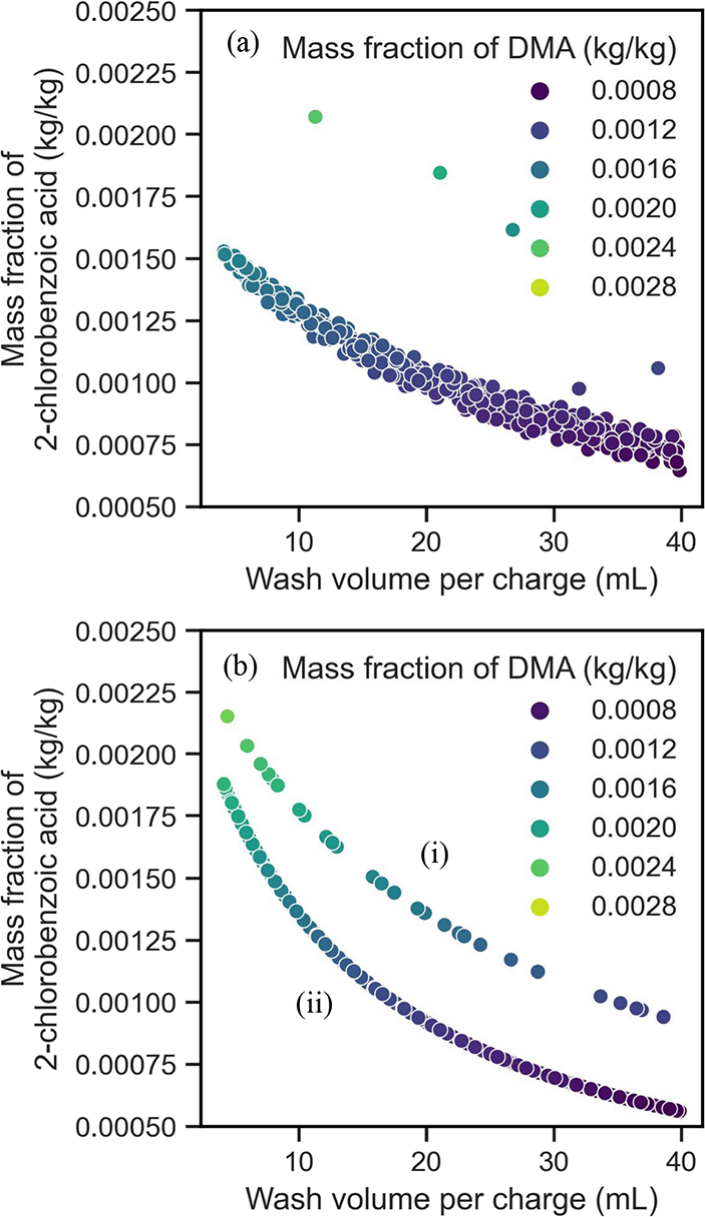
Design space explorations: wash volume against impurity concentration for (a) a single wash cycle and (b) multiple wash cycles for the diglyme-water and cyclohexane system.

Figure 3 shows the results of the two design space explorations conducted, displaying the wash volume against impurity concentration in both figures for experiment 2. Figure 3b shows the effect of multiple washes. When considering a single wash (Figure 3a), the more wash solvent you use, the more effective it is at reducing the impurity concentration. This is in line with what is expected, as well. Figure 3a also shows that after 20 mL (equivalent to 3 cake volumes), the change in impurity concentration is much lower for every milliliters of wash solvent increase. This is a useful finding as it can be used further for optimization and scalability while reducing solvent usage. Figure 3b shows the effect of multiple washes on the impurity concentration, where 3b(i) represents two washes and 3b(ii) represents three washes. This has been set up with a fixed volume of 17 mL for the first wash and a varying time and volume for the subsequent washes. The graph suggests that multiple washes have an effect on the impurity concentration. For the same wash volume, there is a clear reduction in the final impurity concentration. It is also quite clear that a higher wash volume leads to a reduction in impurities. The final concentration of the impurity with an additional wash cycle is also similar to that with only a single wash with a higher wash solvent volume used.

Ottoboni et al*.*^32,33^ demonstrated that small and multiple aliquots of wash solvents improve impurity removal since with multiple washes the back-mixing effect can be minimized when compared to the use of a single large aliquot of wash solvent. As reported by Ottoboni et al*.*,^32,33^ washing the cake with a single aliquot of wash solvent causes a longer contact time between impure mother liquor and clean wash solvent, with risk of impurity migration in the clean wash solvent. Since this model was designed to have instant mixing between mother liquor and washing solvent during washing, the model is not able to predict the intermediate or null back-mixing effect and, therefore, is not capable to distinguish the impurity removal effect due to different washing cycles described by Ottoboni et al.^32,33^

#### 3.4.2 PCM Case Study

**Figure 4:**
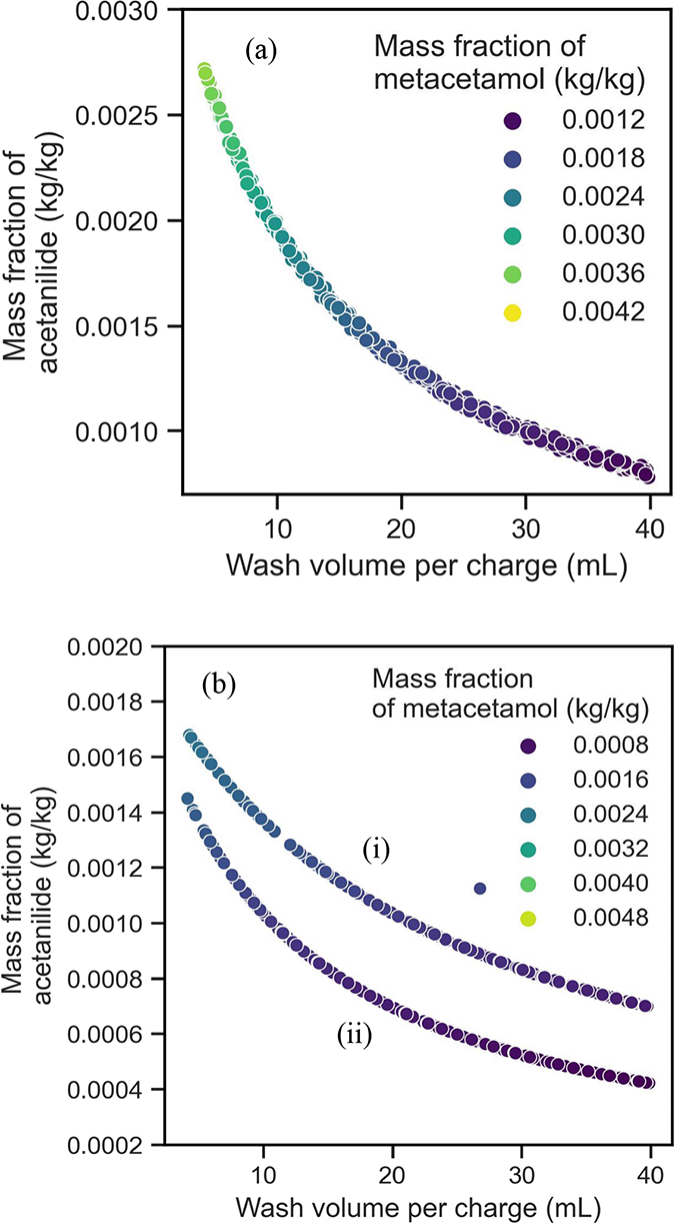
Design space explorations: wash volume against impurity concentration for (a) a single wash cycle and (b) multiple wash cycles for the isoamyl alcohol and dodecane system.

A similar trend is observed for the two design space explorations conducted with the PCM case study (Figure 4). The washing time used for the first washing corresponded to 1s, while the time for the second washing corresponded to 14.22 s. The mass fraction of acetanilide in the initial suspension corresponded to a mass fraction of 0.0015 kg/kg of the total suspension. Even for the PCM case study, after 20 mL of wash solvent used, there is no extra meaningful impurity removal, confirming the experimental evidence reported by Ottoboni et al*.*^32,33^

As also reported in Section 4.4.1, a higher wash volume leads to a reduction in impurities, and this can be done by adding multiple washing stages (Figure 4b).

## 4 Conclusions

To facilitate the process development of APIs without extensive experimental work, a digital tool capable of transferring material property information between unit operations to predict the product attributes in integrated purification processes has been developed.

A mechanistic workflow for the optimization of an integrated filtration and washing model minimized impurities in the isolated cake. This workflow procedure first estimates product and process characteristics (e.g., particle sphericity, porosity, cake and medium resistance, and cake compressibility) using a gPROMS FormulatedProducts Carman-Kozeny filtration model with filtration stopped to dryland. For model validation, a series of experiments were used with MFA and PCM and their related impurities in a series of different crystallization and wash solvents. In general, the estimated cake and filtration parameters using the cake and filtration parameters match the experimental results (cake and medium resistance). In general, the estimated cake compressibility was in the Darcy law range. The model allowed for a quick and relatively accurate calculation of the cake compressibility index, which would have taken much longer to obtain experimentally.

The estimated product and process parameters were then used to simulate filtration and washing using the two modeling approaches, designed to use different washing mechanisms; pure displacement (integrated pressure filter and washing model) or diffusion dispersion (washing model based on MSMPR crystallizer). The filtration Darcy plot and the solvent compositions for the filtrate after filtration and washing were used for model configuration and validation. Overall, the simulated Darcy plots reported in Figure 1 align well with the experimental results, except for the experiment 6 for MFA case, while some discrepancy in the PCM concentration in filtrate collected during wash 1 was observed for experiment 1.

Considering the mass of filtrate removed during the experiments, in some cases the predicted outcome is slightly different when compared to the experimental value: the pressure filter model considers a filtration process exactly stopped to dryland, while during the experiments, human error in estimating the filtration end point can interfere with the accuracy of the results.

When the experimental and predicted composition of filtrate removed during filtration and washing generated with the integrated pressure filter and washing model and the MSMPR model are compared, the pressure filter model is not able to predict the composition well enough due to the washing mechanism approach used (displacement mechanism) and the mechanism assumptions. Instead, to get better simulated washing efficiency, in terms of mother liquor and impurity removal, it is therefore required to simulate a washing as the combination of displacement, diffusion, and dispersion mechanisms, and therefore, the MSMPR washing model is capable with good accuracy to get the final composition of the filtrate after filtration and washing.

The diffusion dispersion model (MSMPR washing model) was then used for design space exploration (using the Global Systems Analysis approach) to identify which washing conditions (wash solvent volume, amount of washing stages, and washing time) reduce the impurity concentration in the final cake after washing. Overall, a strong correlation was observed between the wash solvent volume used and the final purity achieved. In general, a higher volume of washing solvent resulted in a lower amount of residual impurities left in the washed cake. Another outcome obtained from the design space exploration was that there is no difference in the final purity between the use of multiple small aliquots of wash solvent and the use of one large aliquot of wash solvent to wash the cake. This result, which contradicts previous investigations,^32,33^ is due to the assumptions used to design the model.

Future work will be done to consider the dissolution of the solid cake, with and without impurities, with considerations for the nonhomogeneous composition of the cake during washing.

## Data Availability

All data underpinning this publication are openly available from the University of Strathclyde KnowledgeBase at (DOI: 10.15129/2506a2d2-d67c-4be7-a975-60071f675d88).

## Supporting Information Available

Model parameter initial conditions for filtration and washing process HPLC results for mefenamic acid and paracetamol case studies Particle size analysis results for mefenamic acid and paracetamol (PDF)

## Abbreviations

API: active pharmaceutical ingredient
MSMPR: mixed-suspension, mixed-product-removal crystallizer
HPLC: high performance liquid chromatography
PCM: paracetamol
MFA: mefenamic acid
PE: parameter estimation
V: validation
PSD: particle size distribution
THF: tetrahydrofuran
CBA: 2-chlorobenzoic acid
A: acetanilide

## Nomenclature

(cw)I: wash solvent concentration, –
(cw)e: effluent concentration, –
*d*: particle diameter, m
*D*: diameter of the filter chamber, m
*D*_*L*_: axial dispersion coefficient, m^2^ s^–1^
*L*: cake height, m
Pec: overall flow in the cake, m s^–1^
Pep: flow of fluid in proximity of a particle with size *d*, m s^–1^
*u*: superficial velocity of fluid, m s^–1^
us: superficial velocity of wash, m s^–1^
*W* = (*u*_w *t*)/(*L*ε_av) = _w/*v*_u: wash ratio, m^3^ m^–3^
*t*: time, s
**Greek letters**
ε: cake porosity, –
**Subscripts and superscripts**
e: exit of filter cake
i: initial
j: species j
w: inlet wash stream
