# Supplementary material for: Integrated Filtration and Washing Modeling: Optimization of Impurity Rejection for Filtration and Washing of Active Pharmaceutical Ingredients
Source: Org Process Res Dev. 2024 Mar 12;28(4):1089–101. doi: 10.1021/acs.oprd.3c00480 (PMC11036383; doi:10.1021/acs.oprd.3c00480)
Supplement: Supplementary file 1 — op3c00480_si_001.pdf [file op3c00480_si_001.pdf]

Supplementary information
Integrated Filtration and Washing Modelling: Optimization of Impurity Rejection for Filtration and washing of Active Pharmaceutical Ingredients

*Bhavik A. Mehta[1,2], Ekaterina Gramadnikova[1], Cameron J. Brown[*1], Niall A. Mitchell[2], Sara Ottoboni[1,3]*

[1] EPSRC Centre for Innovative Manufacturing in Continuous Manufacturing and Crystallisation, University of Strathclyde, Glasgow, G1 1RD, UK
[2] Siemens Process Systems Engineering Ltd., 26-28 Hammersmith Grove, W6 7HA, London
[3] Department of Chemical and Process Engineering, University of Strathclyde, Glasgow, G1 1RD, UK

Table S1: Parameters used as initial condition for the washing process done after filtration stopped to dryland

**Parameters (initial condition)**	**Unit mass**	**Paracetamol case**	**Mefenamic acid case**
Void fraction	-	0.44	0.3916
Filter media diameter	m	0.027	0.027
Solid mass	-	5.895	4.43
Solid density	kg/m^3	1260	1203
Liquid composition, crystallisation solvent mass fraction	-	0.88	0.93
Liquid composition, API mass fraction	-	0.12	0.07
Liquid composition, wash solvent mass fraction	-	0	0
Crystallisation solvent density	kg/m^3	786 (isopropanol)	806 (2-butanol)
Wash solvent density	kg/m^3	Depend on the solvent selected (see supporting information)	684 (heptane)

HPLC Results

Table S2: HPLC analysis for mefenamic acid and its impurities

Crystallisation solvent	ethyl acetate	diglyme 89%-water 11%	ethyl acetate	ethyl acetate	diglyme 89%-water 11%
Wash solvent 1	10 % Ethyl Acetate 90% Cyclohexane	10 % Diglyme 90% n-Heptane	20 % Ethyl Acetate 80% n-Heptane	21 % Ethyl Acetate 80% n-Heptane	10 % Diglyme 90% Cyclohexane
Wash solvent 2	Cyclohexane	n-Heptane	n-Heptane	n-Heptane	Cyclohexane
Wash solvent 3	Cyclohexane	n-Heptane			
Expt Ref	**1**	**2**	**3**	**4**	**5**
**stream 2**					
solution mass (kg)	0.0309	0.0340	0.0296	0.0308	0.0335
MA (kg)	4.350E-04	1.210E-04	6.582E-04	2.538E-05	1.938E-03
MA (kg/kg solution)	1.406E-02	3.562E-03	2.221E-02	8.246E-04	5.776E-02
CBA (kg)	1.124E-04	1.576E-04	1.314E-04	1.668E-04	3.247E-04
CBA (kg/kg solution)	3.635E-03	4.637E-03	4.435E-03	5.419E-03	9.679E-03
**stream 5**					
solution mass (kg)	1.360E-03	1.194E-03	9.556E-04	1.075E-03	1.496E-03
MA (kg)	1.929E-06	2.530E-05	1.160E-05	5.488E-06	1.114E-05
MA (kg/kg solution)	1.418E-03	2.118E-02	1.214E-02	5.105E-03	7.443E-03
CBA (kg)	2.430E-06	2.785E-06	2.370E-06	3.486E-06	3.072E-06
CBA (kg/kg solution)	1.787E-03	2.332E-03	2.480E-03	3.243E-03	2.053E-03
**stream 6**					
mass wet cake (kg)	4.130E-03	3.359E-03	3.429E-03	3.855E-03	4.425E-03
MA (kg)	1.128E-06	1.224E-06	4.433E-07	1.046E-06	1.275E-06
MA (kg/kg solution)	9.819E-04	1.427E-03	1.058E-03	1.309E-03	1.126E-03
CBA (kg)	1.935E-08	1.033E-07	1.606E-09	3.309E-08	4.268E-08
CBA (kg/kg solution)	1.684E-05	1.204E-04	3.832E-06	4.141E-05	3.767E-05
MA mass total (kg)	3.055E-03	2.775E-03	3.054E-03	3.101E-03	3.677E-03
MA (kg/kg in wet cake)	7.398E-01	8.262E-01	8.907E-01	8.043E-01	8.309E-01

mass solvent (kg)	6.985E-05	2.055E-05	4.822E-05	3.245E-05	6.132E-05
solvent (kg/kg)	1.733E-02	6.245E-03	4.284E-01	8.539E-03	1.428E-02
	1.215E-01	8.375E-02	0	9.359E-02	7.743E-02
	9.827E-01	9.938E-01	5.716E-01	9.915E-01	9.857E-01
MA (kg/kg dry)	7.528E-01	8.314E-01	1.558E+00	8.112E-01	8.429E-01
CBA (kg/kg dry)	1.236E-01	8.428E-02	0	9.440E-02	7.855E-02

Crystallisation solvent	diglyme 89%-water 11%	diglyme 89%-water 11%	diglyme 89%-water 11%	diglyme 89%-water 11%
Wash solvent 1	10 % Diglyme 90% Cyclohexane	10 % Diglyme 90% Cyclohexane	20 % Diglyme 80% n-heptane	10 % Diglyme 90% Cyclohexane
Wash solvent 2	Cyclohexane	Cyclohexane	n-heptane	Cyclohexane
Wash solvent 3	Cyclohexane	Cyclohexane	n-heptane	Cyclohexane
	**6**	**7**	**8**	**9**
**stream 2**				
solution mass (kg)	0.0318	0.0329	0.0311	0.0323
MA (kg)	1.090E-03	1.844E-03	1.311E-03	1.923E-03
MA (kg/kg solution)	3.429E-02	5.603E-02	4.219E-02	5.952E-02
CBA (kg)	1.632E-04	4.845E-05	1.856E-04	2.610E-04
CBA (kg/kg solution)	5.133E-03	1.472E-03	5.976E-03	8.077E-03
**stream 5**				
solution mass (kg)	1.360E-03	1.360E-03	1.194E-03	1.496E-03
MA (kg)	2.799E-05	1.908E-05	1.412E-05	1.635E-05
MA (kg/kg solution)	2.058E-02	1.403E-02	1.182E-02	1.093E-02
CBA (kg)	6.442E-06	3.550E-06	2.082E-06	2.467E-06
CBA (kg/kg solution)	4.736E-03	2.610E-03	1.743E-03	1.649E-03
**stream 6**				
with NMR sample				

mass wet cake (kg)	4.568E-03	4.588E-03	4.514E-03	4.676E-03
MA (kg)	1.460E-06	1.220E-06	1.126E-06	1.453E-06
MA (kg/kg solution)	1.275E-03	1.126E-03	1.034E-03	1.200E-03
CBA (kg)	4.347E-08	3.945E-08	1.462E-08	4.195E-08
CBA (kg/kg solution)	3.796E-05	3.639E-05	1.343E-05	3.464E-05
MA mass total (kg)	3.784E-03	3.840E-03	3.761E-03	3.851E-03
MA (kg/kg in wet cake)	8.283E-01	8.370E-01	8.331E-01	8.236E-01
mass solvent (kg)	6.652E-05	1.594E-04	3.773E-05	1.107E-04
solvent (kg/kg)	1.483E-02	1.847E-02	8.478E-03	2.417E-02
	7.842E-02	7.228E-02	7.920E-02	7.612E-02
	9.852E-01	9.815E-01	9.915E-01	9.758E-01
MA (kg/kg dry)	8.408E-01	8.527E-01	8.402E-01	8.440E-01
CBA (kg/kg dry)	7.960E-02	7.364E-02	7.988E-02	7.800E-02

Table S3: HPLC analysis for paracetamol and its impurities

Crystallization Solvent	Ethanol	Ethanol	Isoamyl Alcohol	Isoamyl Alcohol	Ethanol
Wash Solvent 1	Dodecane	Dodecane	Dodecane	Dodecane	Isopropyl Acetate
Wash Solvent 2	Dodecane	Dodecane	Dodecane	Dodecane	Isopropyl Acetate
Expt Ref	1	2	3	4	5
**R1**					
PCM (mg/ml)	107.8823	108.5982	61.5415	45.5122	115.6179
Meta (mg/ml)	5.3544	5.4206	3.0501	3.4329	4.1392
Acetanilide (mg/ml)	4.9962	5.0407	3.2635	3.5909	4.1191
PCM (g/g)	0.1367	0.1376	0.0760	0.0562	0.1465
Meta (g/g)	0.0068	0.0069	0.0038	0.0042	0.0052
Acetanilide (g/g)	0.0063	0.0064	0.0040	0.0044	0.0052
Mass fraction PCM (g/g solution)	0.1203	0.1210	0.0706	0.0532	0.1278
Mass fraction Meta (g/g solution)	0.0067	0.0068	0.0038	0.0042	0.0052
Mass fraction Aceta (g/g solution)	0.0063	0.0063	0.0040	0.0044	0.0052
Sum (g/g)	0.1333	0.1342	0.0784	0.0618	0.1382
**R2**					
PCM (mg/ml)	99.6883	105.4216	45.7477	23.2599	100.0248
Meta (mg/ml)	4.8455	5.2348	2.4154	1.2314	3.4655
Acetanilide (mg/ml)	4.6508	4.9191	2.8082	2.0009	3.6233
PCM (g/g)	0.1309	0.1384	0.0600	0.0305	0.1183
Meta (g/g)	0.0064	0.0069	0.0032	0.0016	0.0041
Acetanilide (g/g)	0.0061	0.0065	0.0037	0.0026	0.0043
Mass fraction PCM (g/g solution)	0.1157	0.1216	0.0566	0.0296	0.1058
Mass fraction Meta (g/g solution)	0.0063	0.0068	0.0032	0.0016	0.0041

Mass fraction Aceta (g/g solution)	0.0061	0.0064	0.0037	0.0026	0.0043
Sum (g/g)	0.1281	0.1348	0.0635	0.0339	0.1141
**R3**					
PCM (mg/ml)	43.3722	3.0818	20.6050	7.8891	51.0130
Meta (mg/ml)	0.8795	-0.9948	0.8430	0	0.1365
Acetanilide (mg/ml)	1.6936	0.4559	1.6998	1.1026	1.1809
PCM (g/g)	0.0578	0.0041	0.0275	0.0105	0.0586
Meta (g/g)	0.0012	-0.0013	0.0011	0	0.0002
Acetanilide (g/g)	0.0023	0.0006	0.0023	0.0015	0.0014
Mass fraction PCM (g/g solution)	0.0547	0.0041	0.0267	0.0104	0.0554
Mass fraction Meta (g/g solution)	0.0012	-0.0013	0.0011	0	0.0002
Mass fraction Aceta (g/g solution)	0.0023	0.0006	0.0023	0.0015	0.0014
Sum (g/g)	0.0581	0.0034	0.0301	0.0118	0.0569
**R5**					
PCM (mg/ml)	0.0787	0	20.6050	2.7756	71.9359
Meta (mg/ml)	0	0	0.8430	-0.3805	0.8859
Acetanilide (mg/ml)	0.4135	0.2168	1.6998	1.0099	1.8921
PCM (g/g)	0.0001	-0.0002	0.0275	0.0037	0.0827
Meta (g/g)	-0.0016	-0.0016	0.0011	-0.0005	0.0010
Acetanilide (g/g)	0.0006	0.0003	0.0023	0.0013	0.0022
Mass fraction PCM (g/g solution)	0.0001	-0.0002	0.0267	0.0037	0.0764
Mass fraction Meta (g/g solution)	-0.0016	-0.0016	0.0011	-0.0005	0.0010
Mass fraction Aceta (g/g solution)	0.0006	0.0003	0.0023	0.0013	0.0022
Sum (g/g)	-0.0009	-0.0015	0.0301	0.0045	0.0796
**C**					
PCM (mg/ml)	0.9942	0.8055	0.9001	0.9402	1.0227
Meta (g/g)	0	0	0	0	0

Acetanilide (g/g)	0	0	0	0	0
Paracetamol (g/g)	0.9890	0.9890	0.9861	0.9910	0.9869

Crystallization Solvent	Isoamyl Alcohol	Isoamyl Alcohol	Isoamyl Alcohol
Wash Solvent 1	Isopropyl Acetate	Isopropyl Acetate	Isopropyl Acetate
Wash Solvent 2	Isopropyl Acetate	Isopropyl Acetate	Isopropyl Acetate
Expt Ref	7	8	9
**R1**			
PCM (mg/ml)	51.8089	45.0969	49.6343
Meta (mg/ml)	5.1656	4.4453	5.0374
Acetanilide (mg/ml)	4.8028	4.2608	4.7143
PCM (g/g)	0.0640	0.0557	0.0613
Meta (g/g)	0.0064	0.0055	0.0062
Acetanilide (g/g)	0.0059	0.0053	0.0058
Mass fraction PCM (g/g solution)	0.0601	0.0527	0.0577
Mass fraction Meta (g/g solution)	0.0063	0.0055	0.0062
Mass fraction Aceta (g/g solution)	0.0059	0.0052	0.0058
**R2**			
PCM (mg/ml)	39.6334	38.8804	41.1279
Meta (mg/ml)	3.2721	2.9636	3.6100
Acetanilide (mg/ml)	3.4344	3.2063	3.6765
PCM (g/g)	0.0456	0.0447	0.0473
Meta (g/g)	0.0038	0.0034	0.0041
Acetanilide (g/g)	0.0039	0.0037	0.0042

Mass fraction PCM (g/g solution)	0.0436	0.0428	0.0451
Mass fraction Meta (g/g solution)	0.0037	0.0034	0.0041
Mass fraction Aceta (g/g solution)	0.0039	0.0037	0.0042
**R3**			
PCM (mg/ml)	13.5781	14.5362	9.6461
Meta (mg/ml)	0	0	0
Acetanilide (mg/ml)	0.8527	0.9281	0.6019
PCM (g/g)	0.0156	0.0167	0.0111
Meta (g/g)	0	0	0
Acetanilide (g/g)	0.0010	0.0011	0.0007
Mass fraction PCM (g/g solution)	0.0154	0.0164	0.0110
Mass fraction Meta (g/g solution)	0	0	-0.0007
Mass fraction Aceta (g/g solution)	0.0010	0.0011	0.0007
Sum (g/g)	0.0160	0.0173	0.0110
**R5**			
PCM (mg/ml)	21.8582	36.4007	9.6645
Meta (mg/ml)	0.3468	0.9920	0
Acetanilide (mg/ml)	1.3034	1.8362	0.6366
PCM (g/g)	0.0251	0.0418	0.0111
Meta (g/g)	0.0004	0.0011	0
Acetanilide (g/g)	0.0015	0.0021	0.0007
Mass fraction PCM (g/g solution)	0.0245	0.0402	0.0110
Mass fraction Meta (g/g solution)	0.0004	0.0011	0

Mass fraction Aceta (g/g solution)	0.0015	0.0021	0.0007
**C**			
PCM (mg/ml)	0.8310	0.8038	1.0203
Meta (mg/ml)	0	0	0
Acetanilide (mg/ml)	0.0038	0.0041	0.0048
Meta (g/g)	0	0	0
Acetanilide (g/g)	0	0	0
Paracetamol (g/g)	0.9854	0.9861	0.9865

Raw PSD analysis

Table S4: PSD quantiles for raw mefenamic acid and paracetamol (micronised and powder)

Quantile	Mefenamic Acid (µm)	Paracetamol (µm) Micronised	Paracetamol (µm) Powder
D10	40.47	3.73	9.23
D50	94.27	11.7	48.8
D90	204.54	44.8	198

Filtration parameter estimation

Table S5 Parameters used as initial condition for the filtration and washing processes simulated with gPROMS FormulatedProducts.

Initial conditions	Unit measure
System information	
Component	-
Crystal phase	-
Main solute	-
Liquid properties	
Molecular weight	kg/mol
Mass density coefficient	kg/m^3
Mass specific heat coefficient	J/molK
Dynamic viscosity coefficient	Pas
Crystal properties	
Crystal stoichiometry	-
Mass density coefficient	kg/m^3
Mass specific heat coefficient	J/molK
Enthalpy of crystallisation	J/kg
Volumetric shape factor	-
Solubility	
Key component	-
Solubility coefficient	-
Grid parameters	
Number of grid	-
Grid type	
Min particle size distribution	µm
Max particle size distribution	µm
